# Phylogenetic Diversity and Community Assembly of Vascular Plants Along Environmental Gradients in the Western Pamir–Alay: A Case Study From Surkhandarya Province, Southern Uzbekistan

**DOI:** 10.1002/ece3.73374

**Published:** 2026-04-29

**Authors:** Akrom Ibragimov, Bobur Karimov, Zukhuridin Juraev, Dilmurod Makhmudjanov, Shukherdorj Baasanmunkh, Ami Oh, Komiljon Sh. Tojibaev, Hyeok Jae Choi

**Affiliations:** ^1^ Termez State University of Engineering and Agrotechnologies Termez City Uzbekistan; ^2^ Institute of Botany The Academy of Sciences of the Republic of Uzbekistan Tashkent Uzbekistan; ^3^ Department of Biology and Chemistry Changwon National University Changwon Republic of Korea; ^4^ Division of Research Planning and General Affairs Korea National Arboretum Pocheon Republic of Korea

**Keywords:** conservation, endemism, floristic composition, phylogenetic structure, species richness

## Abstract

Biodiversity plays a fundamental role in ecosystem structure, function, and stability, but its response to environmental gradients is poorly understood in Central Asia. We studied plant species richness, phylogenetic diversity, and community phylogenetic structure along elevational, temperature, and precipitation gradients in Surkhandarya province of southern Uzbekistan. Data were compiled from a 5‐year field survey (2020–2024), herbarium records (TASH, ASH, BM, E, H, LE, M, MW, TAD, MOSM), and digital records from the Plantarium database (plantarium.ru), totalling over 64,500 occurrence records. The flora comprises 2202 species across 615 genera and 96 families, including 62 endemics. Species richness and PD exhibited a left‐skewed, hump‐shaped pattern with a peak at 1000–1100 m, while high‐elevation communities were phylogenetically clustered due to environmental filtering. Mid‐elevations showed phylogenetic overdispersion, reflecting the coexistence of distantly related species. Along climatic gradients, species richness peaked at intermediate temperatures (8°C–12°C) and precipitation (400–500 mm), with phylogenetic structure similarly reflecting clustering at extremes and overdispersion at intermediate conditions. Low‐elevation communities were dominated by drought‐ and salt‐tolerant families (Poaceae, Asteraceae, Amaranthaceae), whereas mid‐elevations supported the highest family diversity. Using endemic and Red Book species records (1504 occurrences of 140 species), a proximity‐weighted conservation prioritization identified a minimal set of areas covering 7.43% of the region, with 4.56% requiring expansion beyond existing protected areas to achieve complete species representation.

## Graphical abstract

Using extensive field, herbarium, and database records, we examine patterns of species richness, phylogenetic diversity, and community assembly along elevational and climatic gradients in an understudied region of Central Asia.

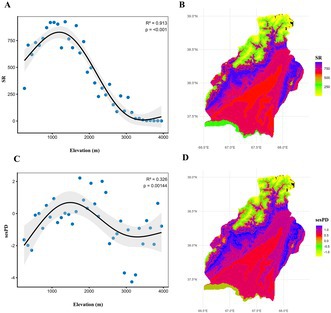

## 1 Introduction

Biodiversity is a critical determinant of ecosystem functioning, stability, and resilience, and its evaluation remains a fundamental objective in ecology and conservation biology. Investigating spatial variation in species diversity along environmental gradients provides insights into underlying ecological and evolutionary processes and informs predictions of climate change impacts, thereby guiding the development of effective conservation strategies (Zhang et al. 2022; Zhao et al. 2023; Li et al. 2025). Traditionally, biodiversity has been assessed using taxonomic measures, such as species or genus richness. To describe spatial variation in biodiversity, Whittaker (1960) proposed three hierarchical levels of diversity: alpha (α), beta (β), and gamma (γ) diversity. Alpha diversity represents species richness within local communities or sampling units, such as vegetation plots (Revermann et al. 2016). Beta diversity describes differences in species composition among communities and reflects spatial turnover along environmental gradients (Koleff et al. 2003; Tuomisto 2010a, 2010b). Gamma diversity refers to the total species richness across larger geographic regions or landscapes (Kier et al. 2005; Brummitt et al. 2021). While these metrics are straightforward and easily interpreted, they provide limited information about functional traits and evolutionary relationships among taxa. Communities with comparable species richness can differ markedly in their evolutionary history and ecological roles (Magurran 2003). Consequently, relying exclusively on taxonomic diversity may underestimate or misrepresent actual patterns of biodiversity (Laity et al. 2015; Miller et al. 2018).

To overcome these limitations, recent studies increasingly incorporate phylogenetic diversity (PD) (Tietje et al. 2023; Qian, Kessler, and Jin 2023; Qian, Zhang, and Jiang 2023; Hughes et al. 2023; Hähn et al. 2025), which accounts for the evolutionary relationships among taxa and represents the total amount of evolutionary history contained within a community (Faith 1992). Community assembly is primarily driven by environmental filtering and competitive exclusion: filtering promotes the coexistence of closely related species with similar traits, producing phylogenetic clustering, while competitive exclusion limits the co‐occurrence of ecologically similar relatives, resulting in phylogenetic overdispersion (Webb et al. 2006; González‐Caro et al. 2014).

Elevational gradients impose sharp environmental changes, generating diverse patterns of species and PD. For example, species richness may decrease while PD increases with elevation (Culmsee and Leuschner 2013), both richness and PD may decline with phylogenetic clustering at higher elevations (Yue and Li 2021), or phylogenetic metrics may display zig‐zag patterns along elevation (Li et al. 2022). These observations indicate that elevational patterns of PD are highly context‐dependent. Elevational gradients provide a powerful natural framework for examining how environmental factors shape floristic composition and evolutionary structure. Although this topic has been widely investigated globally, it remains underexplored in Central Asia. Among the region's mountain systems, the Pamir–Alay Mountains are distinguished by a complex geological history, broad elevational ranges, and pronounced climatic and edaphic heterogeneity. In addition, the Pamir–Alay represents a major biogeographic transition zone linking the Tien Shan to the northeast, the Himalaya to the east, and the Hindu Kush, Kopetdag, Zagros, Alborz, and Caucasus mountain systems to the southwest. As such, the Pamir–Alay particularly its western sector, represented by the Hissar Range, constitutes an exceptional natural laboratory for investigating plant diversity patterns and evolutionary processes. The westernmost part of the Hissar Range lies in southern Uzbekistan, within the Surkhandarya region, where landscapes range from lowland plains in the west to high mountain environments in the east, producing sharp climatic contrasts and a wide diversity of habitats over relatively short distances. This region is characterized by a high concentration of rare, endemic, and relict plant species, many of which are restricted to specific elevational belts or microhabitats (Tojibaev et al. 2022; Yusupov et al. 2025).

Accordingly, during 2020–2024, a grid‐based floristic mapping approach was applied for the first time in Central Asia, using the Surkhandarya region as a case study, to enable a robust and comprehensive assessment of plant diversity, within the framework of which species inventories and descriptive floristic studies were conducted (Juramurodov et al. 2025; Tojibaev et al. 2022). This approach facilitates the standardization, storage, and spatial analysis of floristic data by integrating heterogeneous records collected over different periods into a unified georeferenced database. As a result, a large‐scale dataset comprising more than 64,500 georeferenced records of vascular plant species has been established.

Despite the availability of this standardized grid‐based database, evolutionary‐based analyses remain scarce, not only for the Hissar Range but also across Central Asia more broadly. In particular, the phylogenetic structure of plant communities along elevational gradients in the Surkhandarya region has not yet been adequately examined, despite the substantial volume of available data. Consequently, the relative contributions of environmental filtering, historical processes, and evolutionary constraints to the assembly of plant diversity remain poorly understood. Therefore, the objectives of this study are to: (Q1) assess the floristic composition of plant communities along a broad elevational gradient in the Surkhandarya region; (Q2) examine patterns of PD and phylogenetic structure in relation to elevation and associated environmental factors; and (Q3) how are endemic and rare species spatially distributed, and how do these patterns inform conservation priorities relative to the current protected area network?

## 2 Methods

### 2.1 Study Area

**FIGURE 1:**
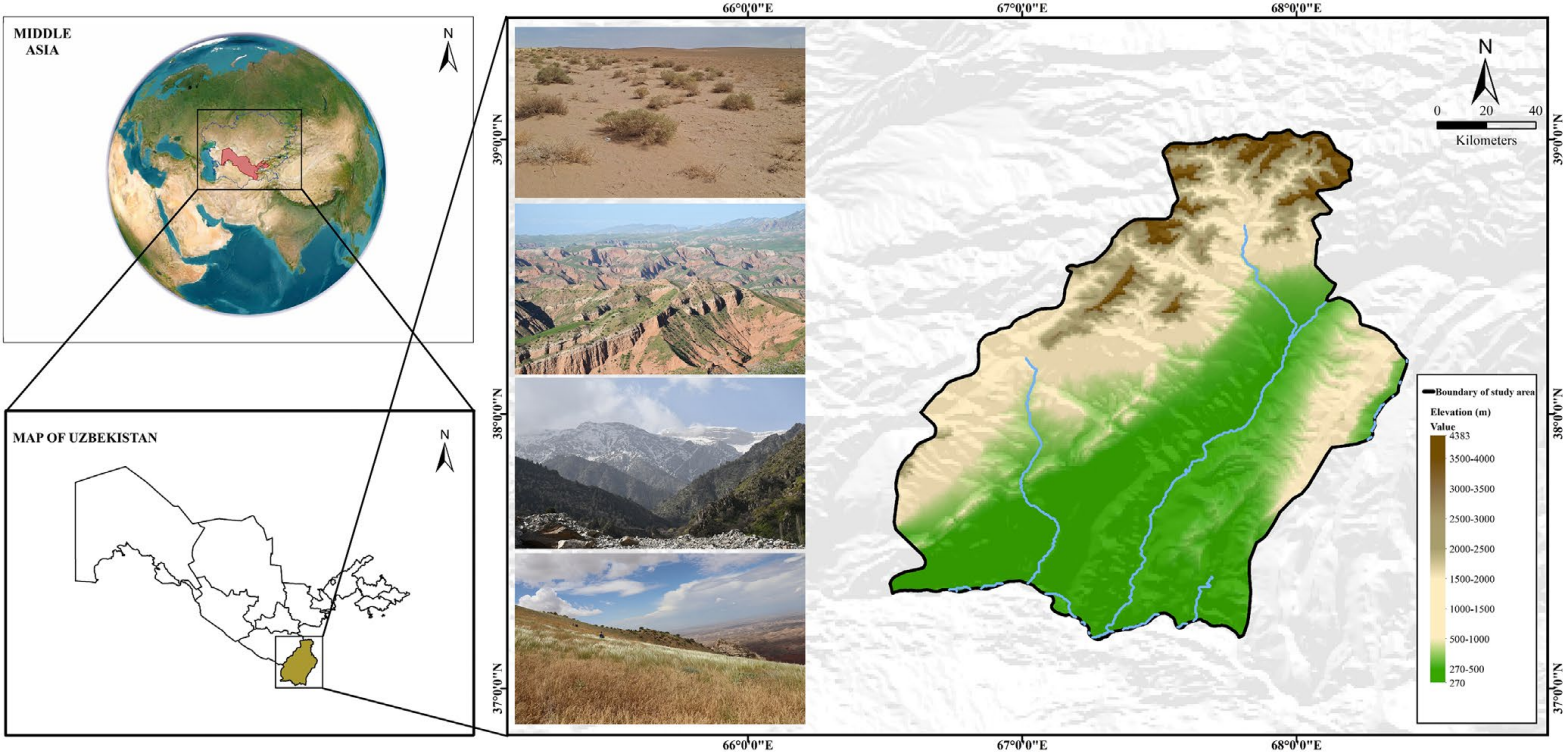
Geographic location, elevation, and major landscape features of the study area (Surkhandarya Province).

Surkhandarya is an administrative region in southeastern Uzbekistan, covering 20,100 km^2^. It borders the Kashkadarya region within Uzbekistan and shares international boundaries with Turkmenistan, Afghanistan, and Tajikistan (Figure 1). The region encompasses a wide range of habitats, from lowland plains to the high mountains of the Hissar–Alay system, with substantial climatic and topographic variation.

### 2.2 Field and Herbarium Data

Plant diversity data were compiled from a 5‐year field survey conducted between 2020 and 2024 and supplemented with herbarium records from TASH, ASH, BM, E, H, LE, M, MW, TAD, and MOSM (VILR), as well as occurrence data from plantarium.ru. In total, more than 64,500 occurrence records were assembled for Surkhandarya Province (Institute of Botany, the Academy of Sciences of the Republic of Uzbekistan 2026) and uploaded to the Grid Mapping of Central Asian Plants database (www.gmoca.org). The most recent period (2020–2024) exhibited a pronounced peak in sampling intensity, with species richness exceeding 1600 species and over 50,000 records, reflecting substantially intensified recent surveys. All species names were standardized according to Plants of the World Online (https://powo.science.kew.org/).

### 2.3 Phylogenetic Reconstruction

The phylogenetic tree was constructed using the R package U.PhyloMaker (Jin and Qian 2023) with scenario S3. This approach utilized the dated mega‐phylogeny GBOTB (Smith and Brown 2018) and its extended version GBOTB.extended.TPL.tre (Jin and Qian 2022) as a backbone. Species absent from the backbone phylogeny were inserted according to their taxonomic hierarchy, with taxa lacking molecular phylogenetic information placed at the genus or family level as polytomies following scenario S3. Of the total species, 795 were directly present in the mega‐tree and inserted accordingly, 1297 species were added at the genus level, and 110 species were incorporated at the family level (Karimov 2026). Phylogenetic trees generated by U.PhyloMaker are very effective and have been widely used in studies on community phylogenetics (Zhang et al. 2021; Qian, Kessler, and Jin 2023; Qian, Zhang, and Jiang 2023; Qian 2025; Zhou et al. 2025).

### 2.4 Phylogenetic Diversity

PD (Faith 1992) was calculated as the total branch length connecting all species within each community. Since PD correlates strongly with species richness (Ding et al. 2021), we used the standardized effect size of PD (ses.PD) calculated via the ‘ses.pd.’ function in the R package Picante (Kembel et al. 2010) with the null model phylogeny. Pool, which generates random communities by sampling species from the regional pool. A total of 999 randomizations were performed to test whether observed PD values significantly deviate from random expectations.

### 2.5 Phylogenetic Structure

Community phylogenetic structure was further assessed using the net relatedness index (NRI) and the nearest taxon index (NTI). NRI is the standardized effect size of the mean pairwise phylogenetic distance (MPD) among co‐occurring species, reflecting overall phylogenetic relatedness. NTI is the standardized effect size of the mean nearest taxon distance (MNTD), indicating relatedness near the phylogenetic tips (Webb 2000; Kraft et al. 2007). Positive values of NRI or NTI indicate phylogenetic clustering, negative values suggest phylogenetic overdispersion, and values near zero indicate randomness. Significant deviations from the null model were considered when values exceeded ±1.96 (Shivaprakash et al. 2018; Ding et al. 2019). Phylogenetic beta diversity along the elevational gradient was quantified using βSOR_phylo, βSIM_phylo, and βNES_phylo, representing total phylogenetic dissimilarity, turnover, and nestedness components, respectively. These indices were calculated using the betapart R package (Baselga and Orme 2012).

### 2.6 Environmental Variables

Elevation, mean annual temperature, and annual precipitation variables were obtained from WorldClim v2.1 at a 30 arc‐second (~1 km) spatial resolution, providing high‐resolution climatic layers based on long‐term averages for the period 1970–2000 (Fick and Hijmans 2017).

### 2.7 Spatial Prioritization of Endemic and Red Book Species Using Grid‐Based Metrics

Species occurrence records were cleaned and projected to UTM Zone 42 N to ensure accurate distance and area calculations. A regular 5 × 5 km grid was generated within the study area, and 1504 occurrence records of 140 endemic and Red Book plant species (threatened and conservation‐priority taxa) of Uzbekistan were assigned to grid cells to create a grid–species presence matrix. Species richness was calculated as the number of species per grid cell, while weighted endemism was computed as the sum of inverse species range sizes. Priority conservation areas were identified using a greedy complementarity‐based algorithm that favored grid cells closer to existing protected areas, resulting in a minimal set of areas achieving complete species coverage. All analyses were conducted in R (R Core Team, 2024) using the sf package for vector data handling (Pebesma 2018), terra for raster processing, and dplyr for data manipulation (Wickham et al. 2023), with visualizations produced using ggplot2 (Hadley 2016).

## 3 Results

### 3.1 Floristic Composition and Diversity of the Surkhandarya Flora

Our floristic inventory indicates that the natural flora of the Surkhandarya Region consists of 2202 wild vascular plant species belonging to 615 genera and 96 families. In total, 62 endemic species belonging to 35 genera and 20 families were recorded in the flora of Surkhandarya, highlighting the region's unique biodiversity. Among these, the most diverse families were Asteraceae with 78 genera (12.68%) and 285 species (12.94%), Fabaceae with 28 genera (4.55%) and 264 species (11.99%), and Poaceae with 67 genera (10.89%) and 176 species (7.99%) (Table 1). Together, the top 15 families represent 437 genera (71.06%) and 1649 species (74.89%) of the total flora, highlighting a concentration of diversity within a limited number of families. Life form analysis showed that hemicryptophytes were predominant, with 987 species, followed by therophytes with 690 species. Cryptophytes, chamaephytes, and phanerophytes comprised 262, 123, and 140 species, respectively.

**TABLE 1:** Dominant plant families in Surkhandarya Province based on genus and species richness.

No.	Family	Number of genera	Percent of genera	Number of species	Percent of species
1	Asteraceae	78	12.68%	285	12.94%
2	Fabaceae	28	4.55%	264	11.99%
3	Poaceae	67	10.89%	176	7.99%
4	Brassicaceae	54	8.78%	129	5.86%
5	Lamiaceae	29	4.72%	125	5.68%
6	Apiaceae	49	7.97%	98	4.45%
7	Boraginaceae	23	3.74%	83	3.77%
8	Caryophyllaceae	19	3.09%	82	3.72%
9	Amaranthaceae	36	5.85%	78	3.54%
10	Ranunculaceae	14	2.28%	70	3.18%
11	Amaryllidaceae	3	0.49%	65	2.95%
12	Liliaceae	3	0.49%	58	2.63%
13	Rosaceae	14	2.28%	47	2.13%
14	Polygonaceae	9	1.46%	45	2.04%
15	Cyperaceae	10	1.63%	44	2.00%
Total		437	71.06%	1649	74.89%

### 3.2 Elevational Patterns of Species Richness and Phylogenetic Diversity

**FIGURE 2:**
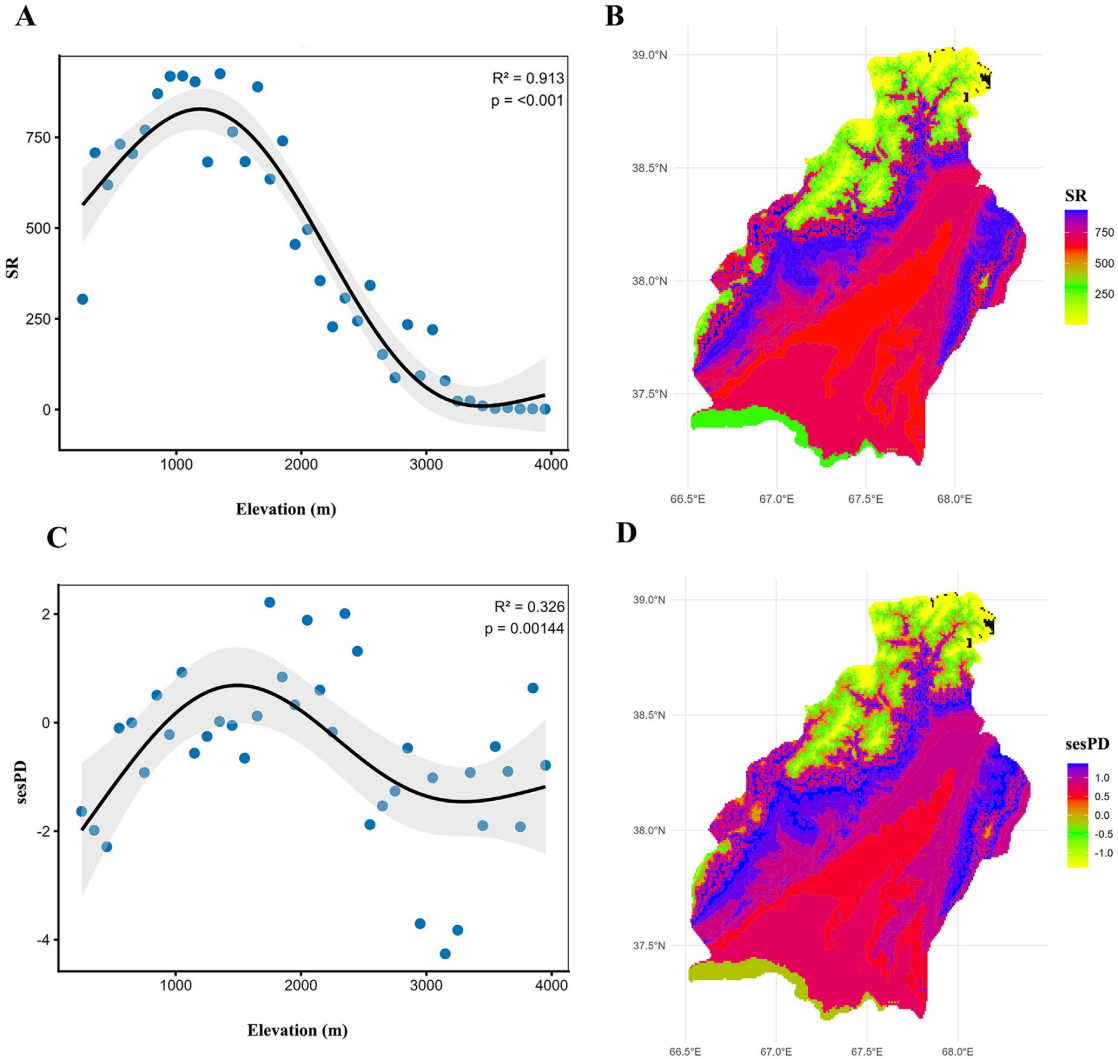
Altitudinal (A, C) and spatial (B, D) patterns of plant species richness and standardized effect size phylogenetic diversity (sesPD).

In the Surkhandarya region, plant species richness along the elevation gradient exhibited a hump‐shaped pattern (*R* ^2^ = 0.913, *p* < 0.001) (Figure 2A,B). At low elevations (200–300 m), species richness was relatively low, with 304 species recorded. As elevation increased, the number of species rapidly rose, reaching a maximum in the 1000–1100 m range (919 species; > 40% of the total flora). Beyond this, species richness gradually declined, ranging from 228 to 496 species at 2000–2300 m, and dropping to only 10–24 species in the highest zones (3300–3500 m).

PD showed a similar trend to species richness, reaching its maximum at mid‐elevations. PD peaked in the 1000–1100 m range (≈21,500) and steadily decreased at both low and high elevations. Above 3000 m, PD sharply declined, with minimal values recorded between 3200 and 3500 m. This elevational pattern was corroborated by sesPD (Figure 2C–D). Negative sesPD values at low and high elevations indicate PD lower than expected under a null model, suggesting phylogenetic clustering or environmental filtering. In contrast, positive sesPD values at mid‐elevations reveal PD levels exceeding random expectations, with the strongest signal shifted slightly upward, peaking between 1700 and 2000 m.

**FIGURE 3:**
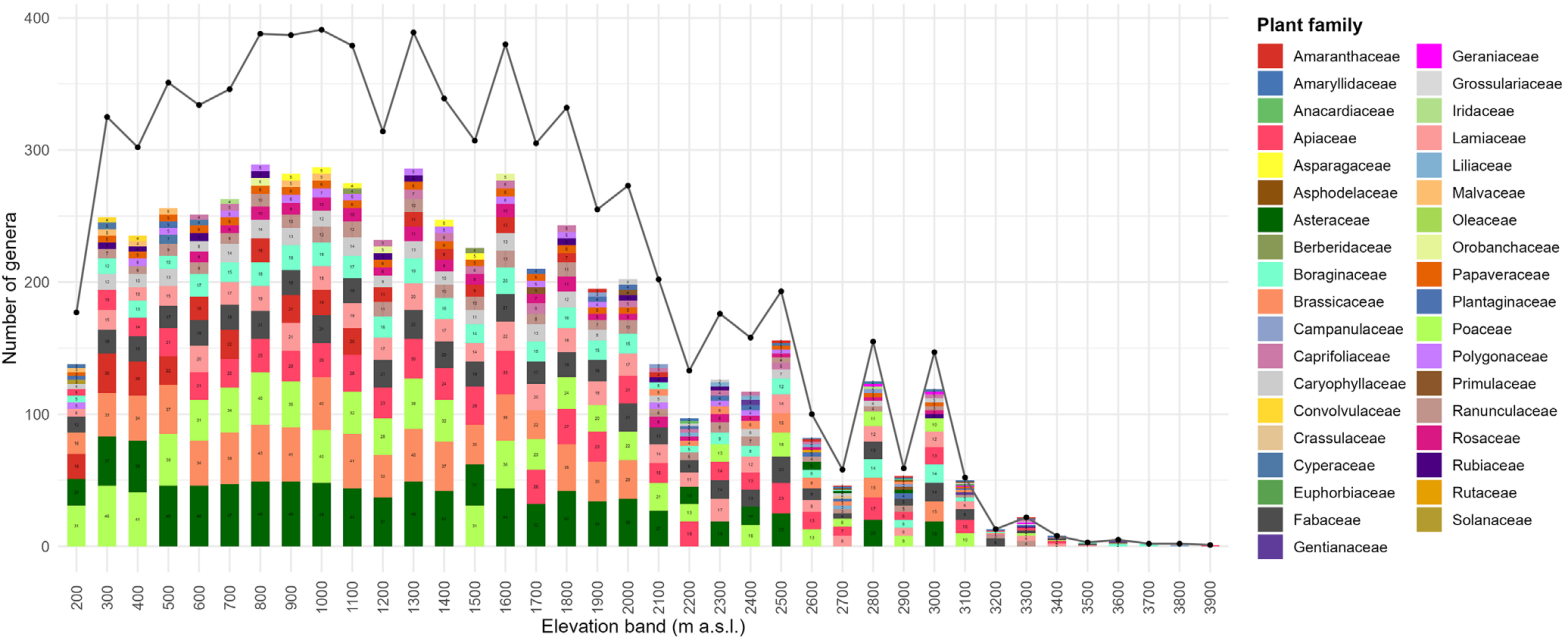
Dominant plant families based on genus richness across elevational bands.

To place these phylogenetic patterns in a floristic context, we examined changes in plant family diversity along the elevational gradient. Plant family diversity exhibited clear and structured variation along the elevational gradient from 200 to 3900 m (Figure 3). Overall, genus richness within families increased from low elevations to mid elevations, followed by a progressive decline toward higher elevations. At lower elevations (200–500 m), plant assemblages were dominated by a small number of highly diverse families. Poaceae, Asteraceae, Brassicaceae, and Amaranthaceae consistently showed the highest genus richness, with Poaceae and Asteraceae reaching early maxima at 300–400 m. Other families, including Fabaceae, Apiaceae, and Lamiaceae, were present but with comparatively lower richness, while several families occurred only sporadically. The mid‐elevation range (600–1600 m) supported the highest overall diversity. During this interval, Asteraceae and Brassicaceae maintained peak or near‐peak genus richness across multiple elevation bins, often exceeding 40 genera. Poaceae remained consistently diverse, while Apiaceae, Fabaceae, Lamiaceae, Boraginaceae, Caryophyllaceae, and Ranunculaceae all showed pronounced increases and reached their maximum richness within this zone. Family composition was most complex at mid elevations, with a larger number of families co‐occurring at moderate to high genus richness. Above ~1700 m, genus richness declined steadily across most families. Low‐elevation families such as Amaranthaceae, Malvaceae, and Cyperaceae decreased sharply in richness or were absent altogether. In contrast, Asteraceae, Apiaceae, Brassicaceae, Fabaceae, and Lamiaceae remained prominent and continued to dominate community composition at higher elevations, although with reduced genus counts. At the highest elevations (> 3000 m), floristic diversity was markedly reduced. Only a limited number of families were recorded, and most were represented by one or a few genera. Apiaceae, Poaceae, Fabaceae, and Lamiaceae were the most consistently present families across high‐elevation bins, while other families such as Boraginaceae, Ranunculaceae, Caryophyllaceae, Gentianaceae, and Primulaceae occurred sporadically.

### 3.3 Elevational Variations in Phylogenetic Structure

**FIGURE 4:**
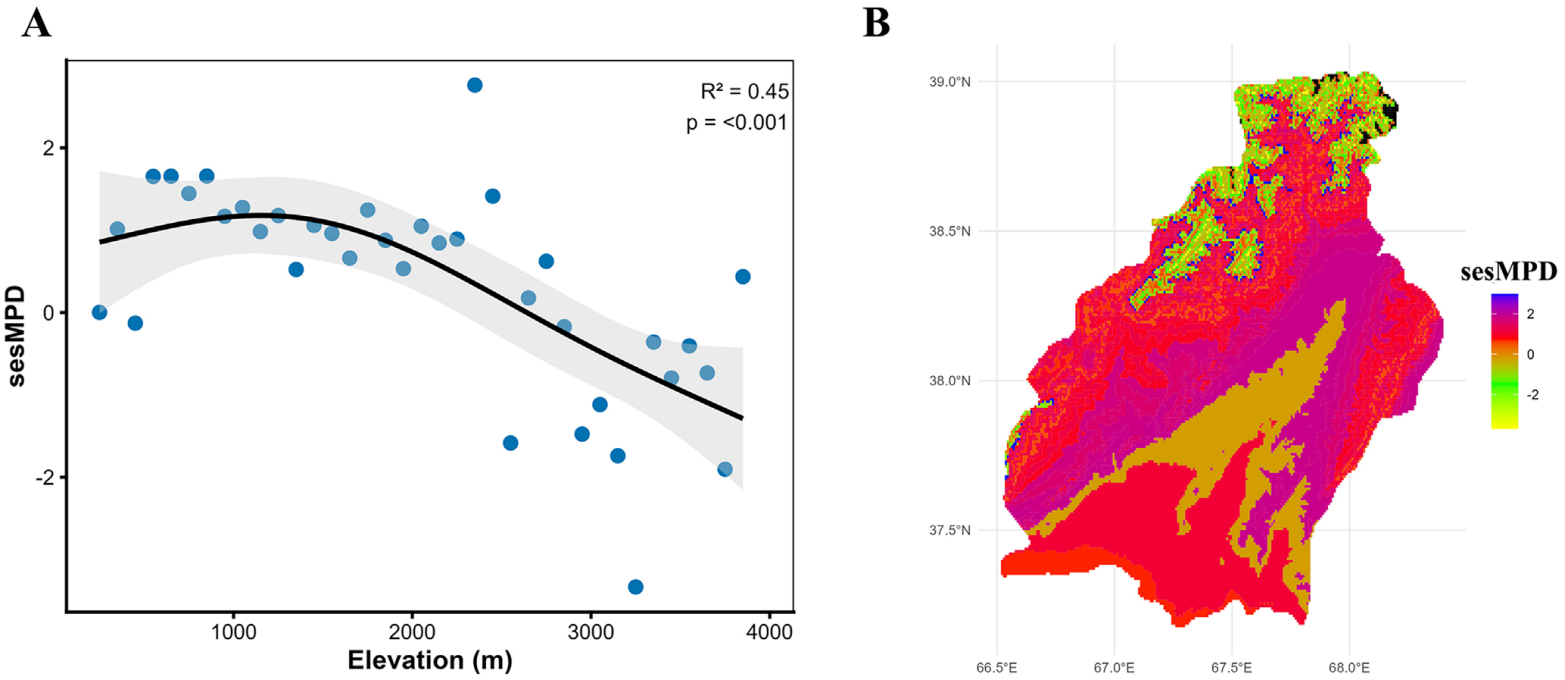
Altitudinal (A) and spatial (B) patterns of phylogenetic structure (sesMPD) in Surkhandarya Province.

**FIGURE 5:**
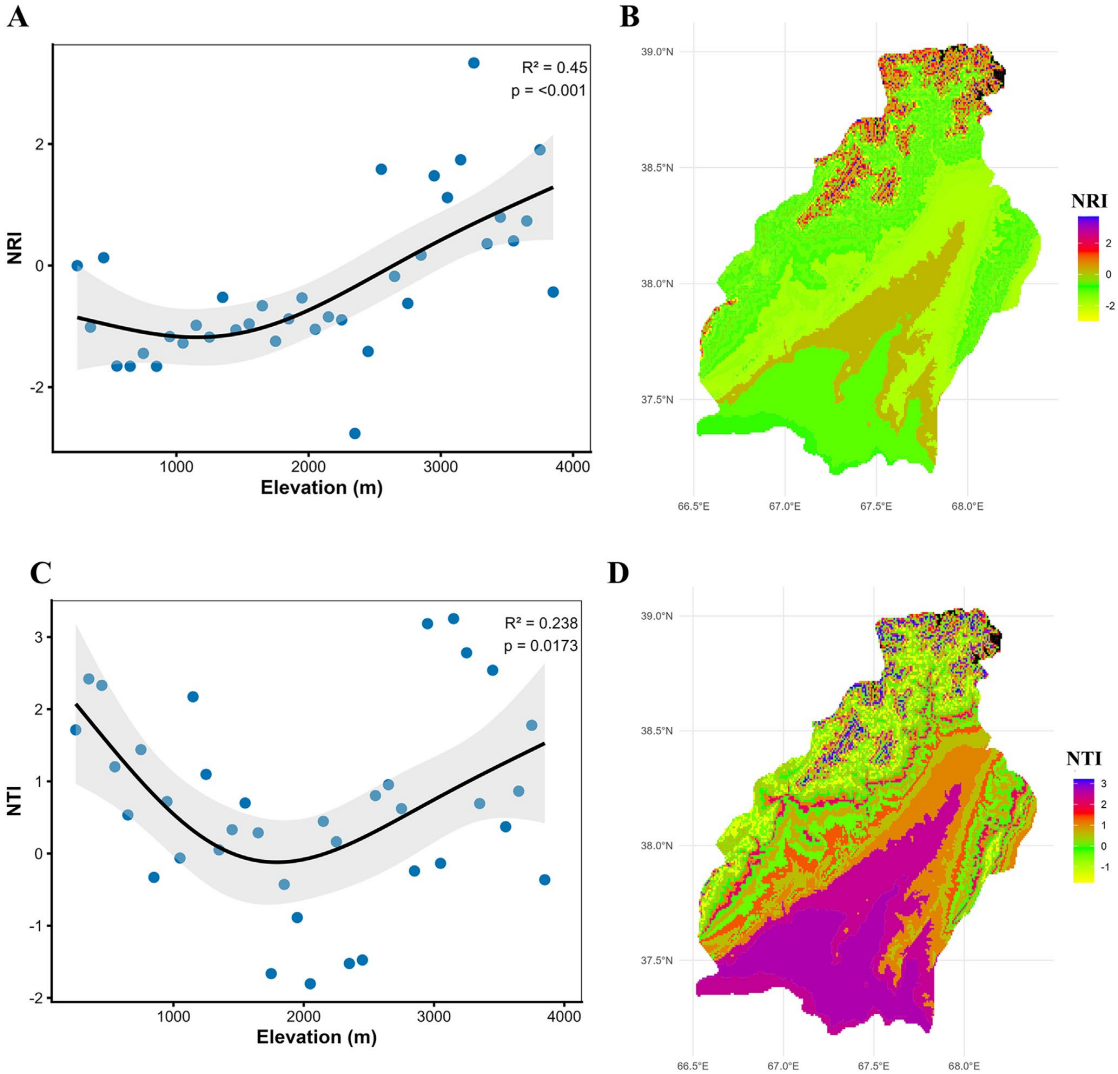
Altitudinal (A, C) and spatial (B, D) patterns of phylogenetic structure (net relatedness index, NRI, and nearest taxon index, NTI).

Phylogenetic structure indices also supported this trend. MPD consistently decreased with increasing elevation (*R* ^2^ = 0.227, *p* = 0.0106), indicating that species within communities become more closely related evolutionarily as elevation rises (Figure 4). In addition, the decline in sesMPD values along the elevation gradient indicates that observed MPD in high‐elevation zones is lower than expected under a random (null) model. This pattern suggests a dominance of phylogenetically closely related species at high elevations and indicates that communities in these areas have a phylogenetically constrained structure. This consistent trend is also clearly reflected in the NRI and NTI (Figure 5). NRI initially showed a slight decline, followed by a pronounced increase with increasing elevation (*R* ^2^ = 0.45, *p* < 0.001), confirming stronger phylogenetic clustering at high elevations. NTI, on the other hand, was high at low elevations, decreased at mid‐elevations (1300–2500 m), and increased again at high elevations. This pattern indicates a tendency for closely related species to co‐occur at low and high elevations, while phylogenetic diversification is stronger at mid‐elevations.

**FIGURE 6:**
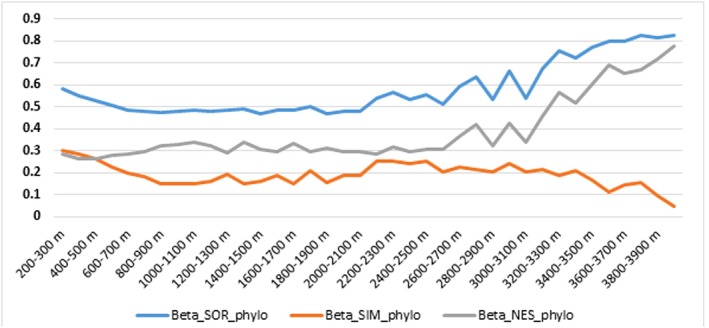
Variation of phylogenetic beta‐diversity components along the elevational gradient, including total phylogenetic dissimilarity (βSOR_phylo), phylogenetic turnover (βSIM_phylo), and phylogenetic nestedness (βNES_phylo).

Phylogenetic beta‐diversity indices also supported this pattern (Figure 6). Results showed that overall phylogenetic beta‐diversity (βSOR_phylo) consistently increased with elevation, ranging from 0.48–0.58 in lowlands and exceeding 0.80 in high‐mountain zones. In contrast, the phylogenetic turnover component (βSIM_phylo) decreased with elevation, falling below 0.1 at high elevations, suggesting limited replacement of phylogenetic lineages. Meanwhile, the phylogenetic nestedness component (βNES_phylo) sharply increased at high elevations, reaching 0.70–0.78, suggesting that high‐elevation communities consist of a phylogenetically reduced subset of species from lower elevations. In other words, floristic communities at high elevations are not composed entirely of new species but rather represent a small, ecologically resilient group of widely distributed phylogenetic lineages from lower elevations.

### 3.4 Changes in Plant Species Richness and Phylogenetic Metrics Across Mean Annual Temperature and Precipitation Gradients

**FIGURE 7:**
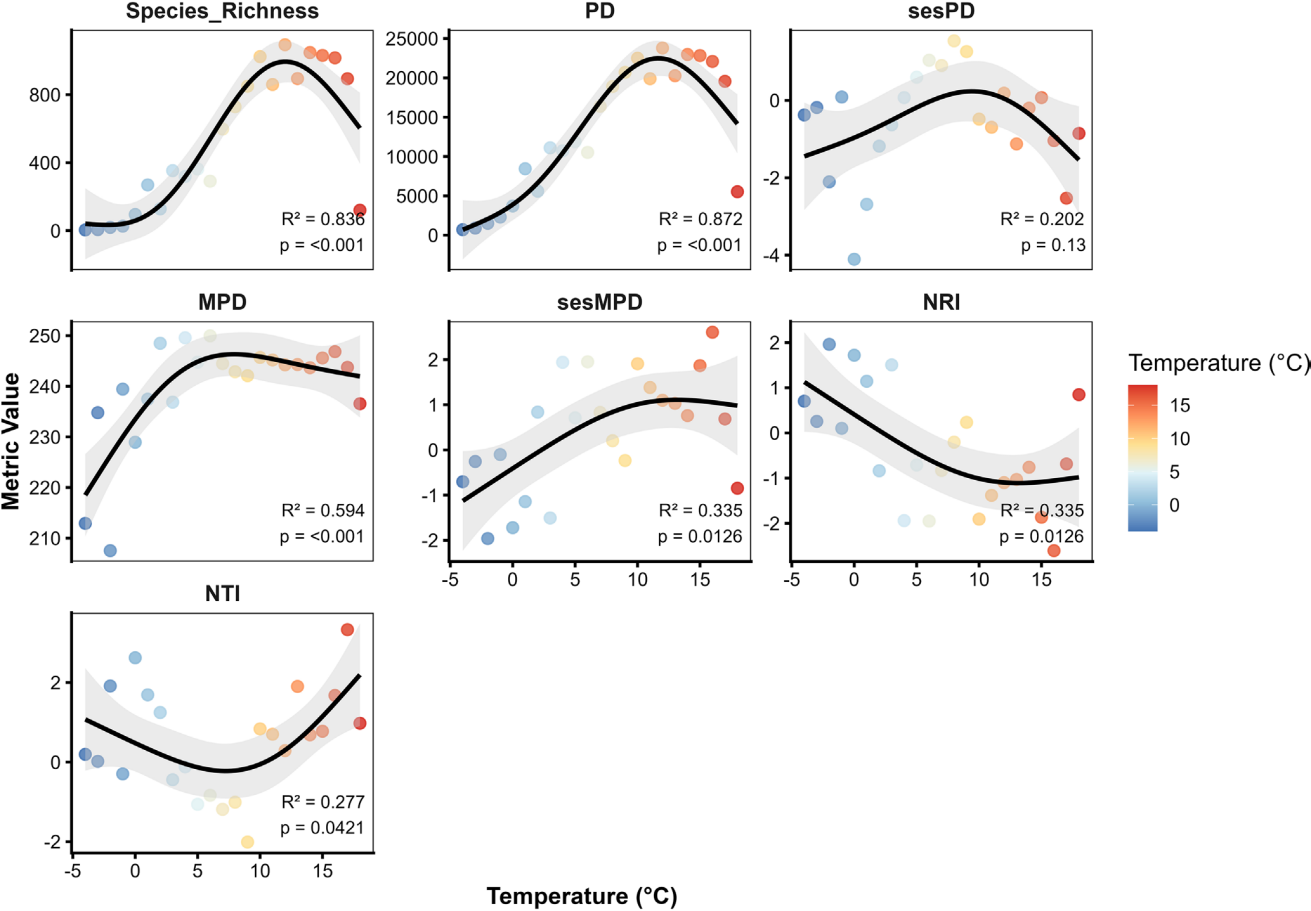
Variation in species richness and phylogenetic diversity indices along the annual mean temperature gradient.

Analysis of plant species richness and phylogenetic indices along the annual mean temperature gradient (−4°C to 18°C) revealed clear nonlinear responses related to climatic conditions (Figure 7). Plant species richness showed a strong hump‐shaped response to temperature, being low at cold extremes, peaking at 8°C–12°C, and declining at higher temperatures (*R* ^2^ = 0.83), indicating optimal conditions at intermediate temperatures. PD increased with temperature, peaking at 12°C–15°C, then declined in warmer zones (*R* ^2^ = 0.87). MPD followed a similar pattern, with mid‐temperature communities composed of more distantly related species, while cold and hot conditions showed stronger phylogenetic clustering (*R* ^2^ = 0.59). Standardized MPD (sesMPD) showed a moderate positive trend at mid to high temperatures (*R* ^2^ = 0.33). NRI decreased with temperature, indicating strong clustering in cold communities. NTI exhibited a U‐shaped pattern: negative at low temperatures, minimal at mid‐temperatures, and rising at high temperatures, reflecting the co‐occurrence of closely related species in extreme temperature zones.

**FIGURE 8:**
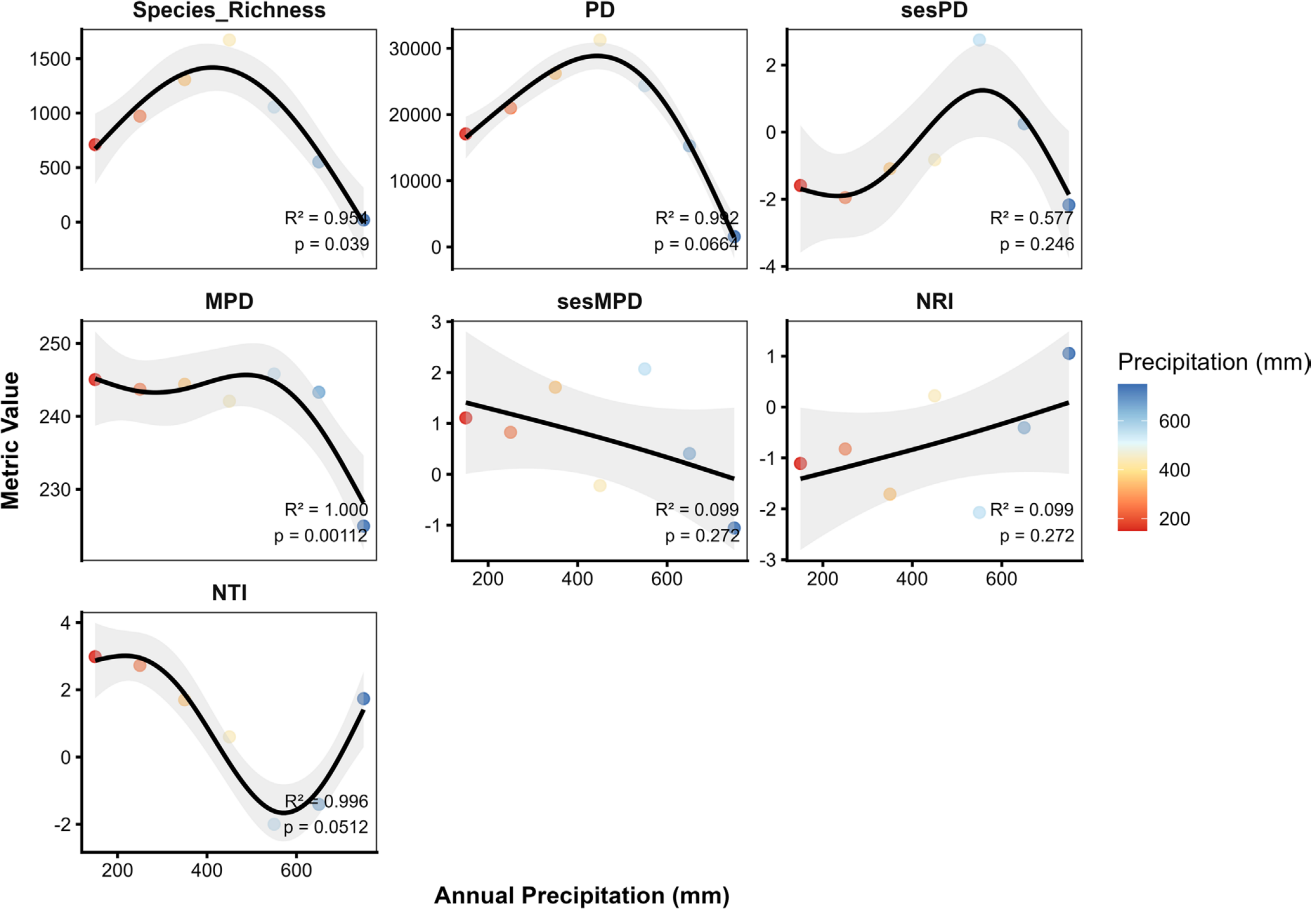
Variation in species richness and phylogenetic diversity indices along the annual mean precipitation gradient.

Along the 100–800 mm precipitation gradient, species richness showed a hump‐shaped pattern, increasing from 711 species at 100–200 mm to a peak of 1671 at 400–500 mm, then sharply declining to 20 species at 700–800 mm (Figure 8). PD followed a similar trend, rising up to 500 mm and declining at higher precipitation. sesPD indicated strong clustering at low precipitation, overdispersion at 500–600 mm, and clustering again at 700–800 mm. MPD and sesMPD mirrored these patterns, showing high phylogenetic distances at mid‐precipitation and reduced distances at the extremes. NRI decreased with precipitation, reflecting a shift from clustering of closely related species at low precipitation to overdispersion at mid‐ to high‐precipitation zones. NTI showed a U‐shaped trend, with strong terminal clustering at low and high precipitation and overdispersion at intermediate levels.

### 3.5 Endemism Hotspots and Conservation Priorities

**FIGURE 9:**
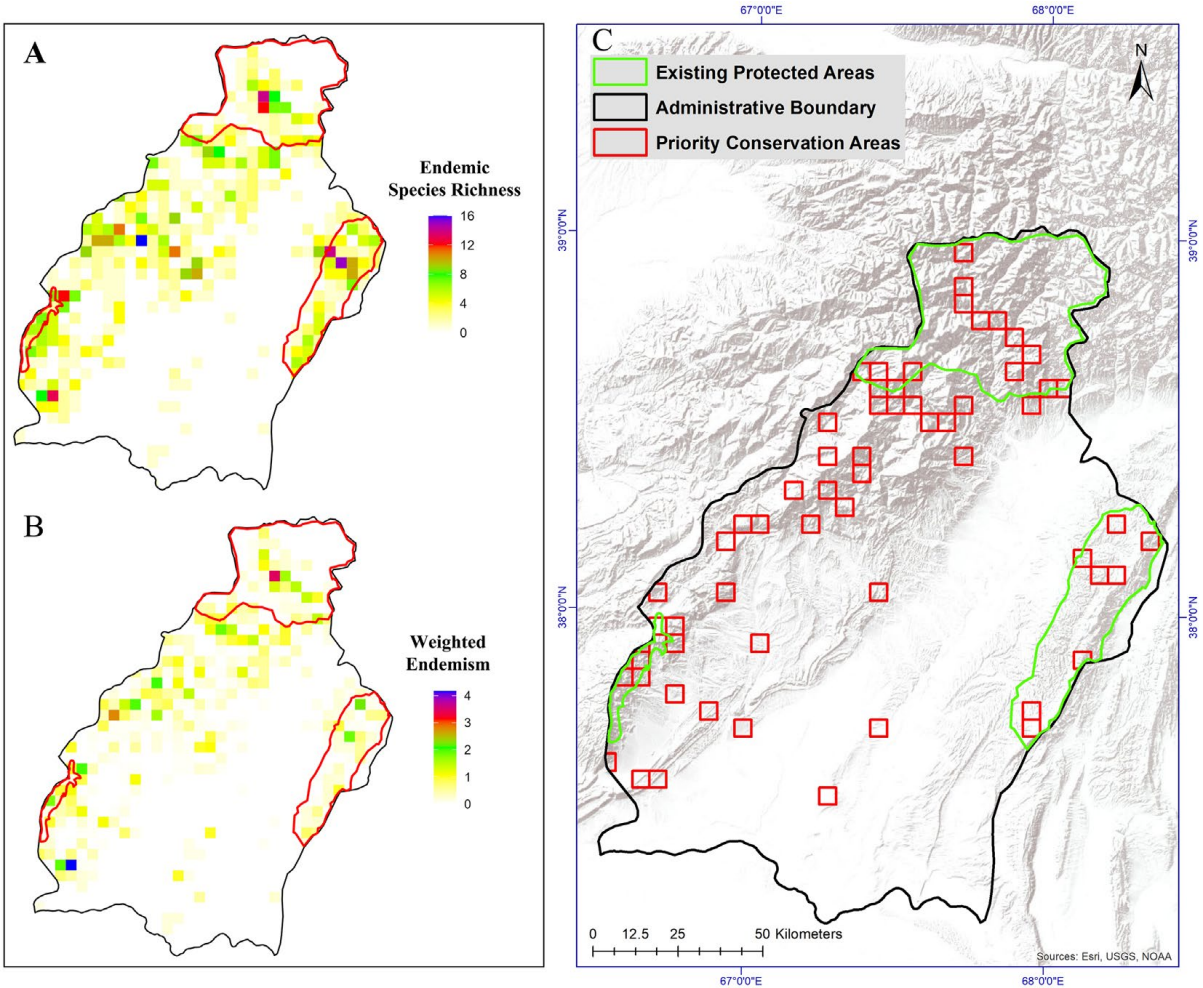
Spatial distribution of conservation‐priority plant species, including endemic and Red Book species: species richness (A), weighted endemism (B), and priority conservation areas identified through proximity‐weighted complementarity analysis (C).

Endemic species richness and weighted endemism showed pronounced spatial heterogeneity across Surkhandarya Region, with hotspots largely concentrated in mountainous areas (Figure 9A,B). Based on 1504 occurrence records of 140 conservation‐priority plant species (including endemic species and species listed in the Red Book of Uzbekistan), the proximity‐weighted complementarity analysis identified a set of priority conservation grid cells that ensure complete representation of all species while minimizing spatial extent (Figure 9C). The total area of Surkhandarya Region is 19,892.3 km^2^, of which existing protected areas cover 3582.0 km^2^ (18.01%). The priority conservation network covers 1477.2 km^2^ (7.43%), including 570.4 km^2^ overlapping with existing protected areas, while 906.8 km^2^ (61.4% of the priority conservation network; 4.56% of the regional area) lies outside the current protected area network. These findings indicate that a substantial portion of priority conservation areas remains outside existing protected areas; strategically located expansions of the protected area system could substantially improve biodiversity representation with minimal additional land requirements.

## 4 Discussion

The documentation of 2202 plant species in Surkhandarya, including 62 endemic taxa, highlights the region as an important reservoir of botanical diversity within the country. In a national context, this richness accounts for more than half of Uzbekistan's total flora, which comprises approximately 4222 species and 301 endemic taxa (Ma et al. 2024). The high floristic richness of Surkhandarya reflects its strong landscape heterogeneity, spanning deserts, steppes, riparian forests, and mountain ecosystems, and its position between the Hissaro–Alai open woodlands and the Badghyz–Karabil semi‐desert ecoregions (Ergashev 1974; Olson et al. 2001). The dominant families in the Surkhandarya flora (Asteraceae, Fabaceae, Poaceae, Brassicaceae, Lamiaceae, Apiaceae, Caryophyllaceae, and Amaranthaceae) exhibit distribution patterns typical of the boreal Holarctic region, reflecting the historical biogeographic connections and evolutionary lineage composition of the regional flora (Malyshev 1987). The predominance of Amaranthaceae, Asteraceae, Brassicaceae, and Poaceae in the region's flora reflects the arid environmental conditions characteristic of the area (Kamelin 2011; Chen et al. 2013). In particular, the high species richness within Amaranthaceae suggests the presence of saline or salt‐affected habitats, indicating adaptation to edaphic stress and xeric conditions. Life form analysis of the Surkhandarya flora indicates that hemicryptophytes and therophytes are dominant; it is a common pattern in many temperate and semi‐arid floras, where these forms are well adapted to climatic stress and seasonal variability (Raunkiaer 1934). Similar dominance of hemicryptophytes and therophytes has been reported in floristic studies of montane and rangeland vegetation (Aipeissova 2017; Alibekov et al. 2025). Overall, the species richness showed a strongly left‐skewed hump‐shaped pattern along the elevational gradient in Surkhandarya, a common trend in plant species richness along extensive elevational gradients (Colwell et al. 2004; McCain and Grytnes 2010).

Our results reveal that PD exhibits a left‐skewed hump‐shaped relationship with elevation, with peak values around 1100 m, mirroring patterns observed for species richness. This congruence supports the widely acknowledged link between PD and species richness, as areas with higher species richness often harbor more phylogenetic lineages (Ding et al. 2021). However, even after accounting for the influence of species richness using sesPD, the left‐skewed hump‐shaped pattern persisted, with a slight shift in peaks toward mid‐elevations (1700–2000 m). Previous studies have reported contrasting elevational patterns of phylogenetic structure in the Himalayas. In the central Himalayas, PD of seed plants showed a strongly hump‐shaped pattern with a peak around 2700 m, although low elevations below 1800 m were not included in the analysis (Liang et al. 2023). In contrast, in the eastern Himalaya, sesPD and NRI displayed zig‐zag patterns along a broader elevational range (100–5300 m), with turning points near 2000 and 4000 m (Li et al. 2022). Phylogenetic structure indices revealed distinct elevational patterns in community assembly.

Together, MPD, sesMPD, NRI, and NTI indicate clear elevational shifts in phylogenetic community structure, characterized by stronger phylogenetic clustering under stressful conditions at both low and high elevations and increased phylogenetic overdispersion at mid‐elevations. At high elevations, this pattern reflects intense environmental filtering imposed by harsh climatic conditions, including low temperatures, shortened growing seasons, and elevated abiotic stress. Similarly, strong phylogenetic clustering at low elevations likely results from filtering under hot and dry conditions, where high temperatures and water limitation restrict community composition to drought‐ and heat‐tolerant lineages (Qian 2018; Luo et al. 2019; Wan et al. 2024; Zhou et al. 2025). Such phylogenetic clustering is consistent with the stress‐dominance hypothesis, which proposes that environmental filters increasingly constrain community assembly as environmental severity intensifies (Weiher and Keddy 1995; Swenson and Enquist 2007).

Phylogenetic overdispersion is often associated with favorable climatic conditions at certain elevations that allow many species to coexist. McCain (2007) suggested that moderate temperature and precipitation at mid‐elevations enhance plant diversity. In our study, areas with an average annual rainfall of about 500 mm and a mean temperature of around 10°C offer suitable environmental conditions that promote phylogenetic overdispersion. This pattern aligns with the ecotone hypothesis, where transitional elevations act as biodiversity hotspots because of the overlap of distinct plant communities (Niu et al. 2024). The elevational variation in family composition further supports the observed phylogenetic patterns. Low‐elevation communities are dominated by drought‐ and salt‐tolerant families such as Poaceae, Asteraceae, and Amaranthaceae, reflecting adaptations to arid plains and semi‐desert conditions (Kamelin 2011; Chen et al. 2013). Phylogenetic clustering at low elevations is likely reinforced by anthropogenic disturbances, as intensified understory grazing and human activities strengthen environmental filtering, favoring closely related species (Luo et al. 2019; Ahmad et al. 2020; Li et al. 2025). In contrast, mid‐ and high‐elevation zones experience lower levels of human disturbance due to reduced accessibility. At high elevations, floristic diversity declines sharply, and only species with specific physiological tolerances such as *Nepeta kokanica*, *Nepeta podostachys*, *Primula algida*, and *Rhodiola heterodonta* persist, indicating strong environmental filtering and limited phylogenetic representation.

The observed phylogenetic and elevational gradients also correspond with patterns of species endemism, suggesting that the same environmental factors shaping community assembly influence the distribution of narrowly ranged taxa. Endemic species, with their limited ranges and specific habitat needs, are highly vulnerable to extinction and serve as important indicators for conservation planning (Lamoreux et al. 2006). In Surkhandarya Province, endemism patterns show that mountainous areas hold the highest conservation value, a trend consistent with other regions of Central Asia and beyond (Noroozi et al. 2018; Ma et al. 2024). Expanding existing protected areas strategically could greatly improve biodiversity representation, as conserving endemic and Red Book species would require protecting only 7.43% of the region, with just 4.56% lying outside current reserves.

## 5 Conclusion

Surkhandarya hosts high plant diversity, including 2202 species and 62 endemics, shaped strongly by elevational gradients. Species richness and phylogenetic diversity peaked at mid‐elevations, while low‐ and high‐elevation communities exhibited strong phylogenetic clustering due to environmental filtering and, at low elevations, anthropogenic disturbances. Mid‐elevation communities were more phylogenetically overdispersed, reflecting greater habitat heterogeneity. These patterns underscore the role of elevation in structuring plant communities and highlight mid‐elevation zones as key reservoirs of both taxonomic and evolutionary diversity, with implications for conservation under climate change. Our conservation prioritization shows that full representation of endemic and Red Book plant species can be achieved by protecting 7.43% of the regional area, with 4.56% requiring expansion beyond existing protected areas.

Future research should integrate functional trait analyses and phylogenomic approaches to further clarify the mechanisms shaping plant community assembly across environmental gradients in the Central Asia region. In particular, two major research directions will be pursued. First, the development of a regional phylogenetic framework for Central Asian vascular plants based on chloroplast genome data, enabling the construction of a comprehensive mega‐phylogeny for the flora of Central Asia. Second, the expansion of the grid‐based floristic mapping approach to document species distribution and diversity patterns across Central Asia. Integrating these large‐scale distribution datasets with phylogenetic information will allow the combined analysis of phylogeny, species distributions, and biodiversity patterns, improving our understanding of regional biogeography and supporting conservation planning under ongoing climate change.

## Author Contributions

**Akrom Ibragimov:** data curation (equal), resources (equal), writing – review and editing (equal). **Bobur Karimov:** methodology (equal), writing – original draft (equal), writing – review and editing (equal). **Zukhuridin Juraev:** methodology (equal), writing – original draft (equal). **Dilmurod Makhmudjanov:** data curation (equal), resources (equal), writing – original draft (equal), writing – review and editing (equal). **Shukherdorj Baasanmunkh:** funding acquisition (equal), project administration (equal), writing – review and editing (equal). **Ami Oh:** funding acquisition (equal), project administration (equal), writing – review and editing (equal). **Komiljon Sh. Tojibaev:** conceptualization (equal), supervision (equal), writing – original draft (equal), writing – review and editing (equal). **Hyeok Jae Choi:** funding acquisition (equal), project administration (equal), writing – review and editing (equal).

## Funding

This work was supported by the State Program “Digital Nature: Development of a Digital Platform for the Flora of Central Uzbekistan” (2025–2029), implemented by the Institute of Botany of the Academy of Sciences of the Republic of Uzbekistan. The study was also conducted within the framework of the CABCN and Central Asia Green Road Project III, supported by the Korea National Arboretum (Project No. KNA1‐249‐25‐2). Additional support was provided by the fundamental research project A‐FA‐2021‐427, “Taxonomic Revision of Polymorphic Families in the Flora of Uzbekistan,” as well as by the project “Bio‐climatic and Geospatial Analysis of the Resource Potential of Medicinal Plants of the Republic: Patterns of Adaptation and Distribution (a Case Study of the Genus *Ferula* L.)” (Project No. FL‐9524115063).

## Conflicts of Interest

The authors declare no conflicts of interest.

## Data Availability

All data supporting the findings of this study are included within the article. Additional datasets, including the plant species distribution dataset (occurrence records, species list, endemic species, and threatened species) and the phylogenetic tree dataset for the Surkhandarya Region, Uzbekistan, are publicly available in the Zenodo repository at https://doi.org/10.5281/zenodo.18979451 and https://doi.org/10.5281/zenodo.18981373.
